# Bacterial xylose isomerases from the mammal gut Bacteroidetes cluster function in *Saccharomyces cerevisiae* for effective xylose fermentation

**DOI:** 10.1186/s12934-015-0253-1

**Published:** 2015-05-17

**Authors:** Bingyin Peng, Shuangcheng Huang, Tingting Liu, Anli Geng

**Affiliations:** School of Life Sciences and Chemical Technology, Ngee Ann Polytechnic, Singapore, Singapore; School of Chemical Engineering and Pharmacy, Key Laboratory for Green Chemical Process of Ministry of Education, Wuhan Institute of Technology, Wuhan, 430073 Peoples Republic of China

**Keywords:** Xylose isomerase, Bacteroidetes, *Alistipes*, *Bacteroides*, Mammal gut, Microbe, Adaptive evolution, *Saccharomyces cerevisiae*, Xylose fermentation, Cellulosic ethanol

## Abstract

**Background:**

Xylose isomerase (XI) catalyzes the conversion of xylose to xylulose, which is the key step for anaerobic ethanolic fermentation of xylose. Very few bacterial XIs can function actively in *Saccharomyces cerevisiae*. Here, we illustrate a group of XIs that would function for xylose fermentation in *S. cerevisiae* through phylogenetic analysis, recombinant yeast strain construction, and xylose fermentation.

**Results:**

Phylogenetic analysis of deposited XI sequences showed that XI evolutionary relationship was highly consistent with the bacterial taxonomic orders and quite a few functional XIs in *S. cerevisiae* were clustered with XIs from mammal gut Bacteroidetes group. An XI from *Bacteroides valgutus* in this cluster was actively expressed in *S. cerevisiae* with an activity comparable to the fungal XI from *Piromyces* sp. Two XI genes were isolated from the environmental metagenome and they were clustered with XIs from environmental Bacteroidetes group. These two XIs could not be expressed in yeast with activity. With the XI from *B. valgutus* expressed in *S. cerevisiae*, background yeast strains were optimized by pentose metabolizing pathway enhancement and adaptive evolution in xylose medium. Afterwards, more XIs from the mammal gut Bacteroidetes group, including those from *B. vulgatus*, *Tannerella* sp. 6_1_58FAA_CT1, *Paraprevotella xylaniphila* and *Alistipes* sp. HGB5, were individually transformed into *S. cerevisiae*. The known functional XI from *Orpinomyces* sp. ukk1, a mammal gut fungus, was used as the control. All the resulting recombinant yeast strains were able to ferment xylose. The respiration-deficient strains harboring *B. vulgatus* and *Alistipes* sp. HGB5 XI genes respectively obtained specific xylose consumption rate of 0.662 and 0.704 g xylose gcdw^−1^ h^−1^, and ethanol specific productivity of 0.277 and 0.283 g ethanol gcdw^−1^ h^−1^, much comparable to those obtained by the control strain carrying *Orpinomyces* sp. ukk1 XI gene.

**Conclusions:**

This study demonstrated that XIs clustered in the mammal gut Bacteroidetes group were able to be expressed functionally in *S. cerevisiae* and background strain anaerobic adaptive evolution in xylose medium is essential for the screening of functional XIs. The methods outlined in this paper are instructive for the identification of novel XIs that are functional in *S. cerevisiae*.

**Electronic supplementary material:**

The online version of this article (doi:10.1186/s12934-015-0253-1) contains supplementary material, which is available to authorized users.

## Background

Bio-ethanol, currently blended in gasoline, is primarily produced from microbial fermentation of food-based sugars, such as corn starch and cane sugar. Bioethanol derived from food-based substrates is unsustainable as it competes with our food chain and may cause human starvation. On the other hand, ethanol production from the abundant lignocellulosic biomass, such as agricultural wastes and forest waste, promises a sustainable green fuel. However, effective conversion of xylose, the second abundant sugar after glucose in lignocellulosic biomass, is necessary to make this process more economically feasible and efficient [1]. *Saccharomyces cerevisiae* is an attractive host to produce biofuels and industrial chemicals from lignocellulosic biomass due to its robustness and fast fermentation rate [2]. However, it cannot naturally utilize xylose [3]. Xylose isomerase (XI) naturally catalyzes the bacterial conversion of xylose to xylulose, which is further metabolized to ethanol through central metabolic pathways. One of the essential metabolic engineering modifications for effective xylose fermentation by *S. cerevisiae* is expressing an XI with high activity. However, the species boundary makes most of eubacterial xylose isomerases fail to be functionally expressed in yeast.

Besides XI pathway, xylose reductase (XR) and xylitol dehydrogenase (XDH) pathway from *Pichia stipitis* (*Scheffersomyces stipitis*) was also engineered in *S. cerevisiae* for the construction of xylose-fermenting yeast [4–7]. In XI pathway, xylose is firstly isomerized into xylulose through one-step catalysis by XI and xylulose was then phosphorylated into xylulose 5-phosphate by xylulokinase. The latter is the intermediate metabolite of pentose phosphate pathway and can be further converted into ethanol through glycolysis [6]. In XR and XDH pathway, xylose is firstly reduced into xylitol by XR, and xylitol is then oxidized into xylulose by XDH [1]. This pathway has been successfully constructed in *S. cerevisiae*; however, xylitol accumulation is a problem. This is caused by the uncoupled cofactor consumption of NADPH-preferred XR and NAD^+^-preferred XDH [5]. Although anaerobic xylose-fermenting yeast was successfully constructed by the overexpression of NADH-preferred XR or NADP^+^-preferred XDH generated through protein engineering [1, 8], xylitol accumulation was still a problem and the specific xylose consumption was not as efficient as XI expressed strains [9–11]. Therefore, xylose isomerase pathway is still the method of choice for the construction of efficient xylose-fermenting yeast.

XI commonly exists in bacteria. However, because of unknown molecular barrier between yeast and bacteria, most Proteobacteria XI could not be expressed in *S. cerevisiae* functionally. It was found that XIs from *Escherichia coli*, *Bacillus subtilis*, *and Streptomyces rubiginosus* were expressed as the insoluble form in *S. cerevisiae* [12–14]. XI from *Thermus thermophilus* was firstly reported to be actively expressed in *S. cerevisiae*. However, its optimal reaction temperature was 85 °C, and at 30 °C it only demonstrated about 4 % of the maximum activity, which was insufficient for fast xylose consumption [4]. The first reported XI that guaranteed effective xylose fermentation in *S. cerevisiae* at 30 °C was from the anaerobic fungus *Piromyces* sp. E2, and the recombinants generated from XI gene transformation and adaptive evolution consumed xylose at the specific rate of 0.18 to 1.87 g^−1^ xylose g^−1^ biomass h^−1^ [6, 7, 15]. XIs from another anaerobic fungus *Orpinomyces* sp. ukk1 [16], several *Bacteroid* bacteria [17, 18], *Clostridium phytofermentans* [19], *Ruminococcus flavefaciens* [20] and *Prevotella ruminicola* [21] have also been successfully used to construct xylose-fermenting yeast. Information on the active XIs in yeast is scattered, and it would be instructive to rational selection of XIs to efficiently construct xylose-fermenting yeast if the evolutionary relationship among the active XIs can be elucidated.

In this study, phylogenetic analysis of the active XIs was conducted and it was found that majority of the reported active XIs were clustered in mammal gut Bacteroidetes group. This suggests that other XIs clustered in this group might also be able to function in *S. cerevisiae*. In order to confirm this, four different deposited XI genes from mammal gut Bacteroidetes group were tested for their functional expression in yeast. In addition, two XIs were cloned from snail manure metagenomic DNA by degenerate PCR and thermal asymmetric interlaced (TAIL) PCR, and they were used as the reference. The two cloned XIs were clustered with XIs from environmental Bacteroidetes bacteria. As expected, only XIs from the mammal gut Bacteroidetes group were expressed in *S. cerevisiae* with activity. Through combined optimization of xylose metabolic pathways and adaptive evolution in xylose medium, recombinant yeast strains containing mammal gut Bacteroidetes XIs were generated and all of them showed efficient xylose fermentation.

## Results and discussion

### Sequence analysis and cloning of xylose isomerase (XI) from environmental mega-genome

**Fig. 1:**
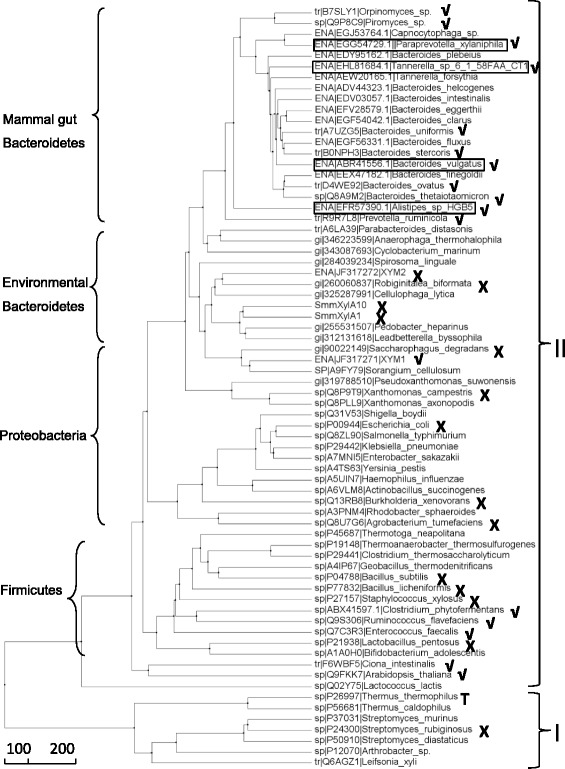
Phylogenetic tree analysis of amino acid sequences of Class I & II xylose isomerases: √, active in *Saccharomyces cerevisiae* at 30 °C; ×, not active at 30 °C for xylose fermentation when expressed in *Saccharomyces cerevisiae* [6, 12–14, 17–21, 23, 29–31]. The sequences were aligned by MAFFT algorithm with default setting and the tree was calculated by average distance using BLOSUM62 through software Jalview (http://www.jalview.org)

With the fast development of genome-sequencing technique, increasing numbers of XI sequences were deposited in public nucleotide/protein databases, such as Genbank, ENA and Uniprot. For the exploration of XIs that are potentially functional in yeast, XI sequences were retrieved and their phylogenetic relationship was analyzed. XIs were grouped into two classes (class I and class II) [22]. Class I XIs are distinguished from class II XIs by having a shorter N-terminal, and *T. thermophilus* XI, the first XI actively expressed in *S. cerevisiae*, belongs to class I. However, another class I enzyme from *S. rubiginosus* was misfolded when expressed in yeast. Class II represents a large number of XI families, and the relationship of XIs in phylogenetic tree is highly consistent with the taxonomic orders (Fig. 1). However, only a few of them are active in yeast. Currently, most of the active XIs in yeast belong to Class II. The phylogenetic tree shows that XI expressed actively at 30 °C in yeast are in three branches, Bacteroidetes, *C. phytofermentans*-*R. flaverfaciens* and higher eukaryotes (*Ciona* and *Arabidopsis*) (Fig. 1), and majority of the active XIs are from gut bacteria (or fungi) of mammals. This suggests that the active expression of microbial xylose isomerase in *S. cerevisiae* might be related to the diverse evolutionary events of mammalian gut commensals where xylan-degrading microorganisms are abundant [21].

Among the active XIs in yeast, XIs originated from the phylum Bacteroidetes represent a significant number. XIs from gut Bacteroiedetes of mammals are categorized into a group different from environmental Bacteroidetes XIs, and XIs from fungi, *Piromyces* and *Orpinomyces*, are also clustered in this group. Therefore, it is very likely that other XIs clustered in this group might also be able to function in *S. cerevisiae*. Based on this hypothesis, in this study, the deposited gene sequence encoding XIs from *Bacteroides vulgatus*, *Alistipes* sp. HGB5, *Paraprevotella xylaniphila*, and *Tannerella* sp. 6_1_58FAA_CT1 from the mammal gut Bacteroidetes group were selected and tested for the construction of xylose-fermenting yeast.

In order to isolate novel functional XI genes in yeast, XI sequences from metagenomic DNA of snail manure and forest soil were directly amplified through degenerated PCR and TAIL-PCR. Interestingly, degenerated PCR products of snail manure sample and forest soil sample shared the same sequences, and the deducted amino acid sequences were related to environmental Bacteroidetes group (Fig. 1). Eleven unique sequences in total were amplified, but all were clustered in environmental Bacteroidete*s* group (data not shown). The upstream and downstream sequences of two degenerated products (SmmXylA1 and SmmXylA10) were amplified successfully through TAIL-PCR. Based on the sequencing results, the full-length genes of SmmXylA1 and SmmXylA10 (SEQ. A1 and SEQ. A2) were amplified directly from metagenomic DNA of snail manure sample and were also tested for their functional expression in yeast.

### Expression of the xylose isomerase from two clades of Bacteroidetes xylose isomerases

**Fig. 2:**
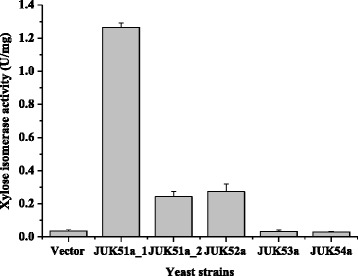
Xylose isomerase activities in the strains harboring empty vector (Vector), or xylose isomerases genes from *Bacteroides vulgatus* (JUK51a_1 and JUK51a_2), *Piromyces* sp. E2 (JUK52a) and snail manure/soil metagenomic DNA (JUK53a; JUK54a)

*B. vulgatus* is a xylan-degrading bacterium habituating in human gastrointestinal tract, and its genome sequence has been released [23]. *B. vulgatus* XI shares 81 % identical amino acid sequence and 38 % identical nucleic sequence with *Piromyces* sp. E2 XI. The full length gene was directly synthesized according to the deposited sequence under EMBL-ENA accession No. ABR41556.1 without codon usage optimization so that the potential of the original XI in its functional expression in *S. cerevisiae* can be evaluated. *B. vulgatus* XI gene was fused with *TDH3* promoter and *PGK1* terminator. In addition, *B. vulgatus* XI gene, *Piromyces* sp. E2 XI (EMBL:AJ249909.1) gene and the two XI genes cloned from metagenomic DNA were cloned between *TEF1* promoter and *CYC1* terminator. Notably, only *B. vulgatus* and *Piromyces* sp. E2 XIs were expressed with activities, and the strains harboring the metagenomic XI genes and the reference strain carrying the empty vector almost showed no activity (Fig. 2).

Active expression of *B. vulgatus* XI in *S. cerevisiae* is expected because its amino acid sequence is close to those of XIs from *Piromyces* sp. E2, *Bacteroides stercoris* [18] and *P. ruminicola* [21], which are all clustered with the XIs from mammal gut Bacteroidetes group. Interestingly, although the original *B. vulgatus* XI gene sequence had a low value (0.146) of codon adaptation index (CAI) to *S. cerevisiae*, the demonstrated activity of *B. vulgatus* XI was significantly high (Fig. 2), revealing the potential function of this XI in *S. cerevisiae*. While *B. vulgatus* XI under the promoter of *TDH3* (JUK51a_1) displayed much higher activity than *Piromyces* sp. E2 XI under *TEF1* promoter (JUK52a), under *TEF1* promoter *B. vulgatus* XI (JUK51a_2) and *Piromyces* sp. E2 XI (JUK52a) exhibited comparable activities (Fig. 2). The fact that *TDH3* promoter is a stronger promoter than *TEF1* promoter [24] might contribute to the high XI activity in JUK51a_1. For the development of background strain, strain JUK51a_1 harboring *B. vulgatus* XI under *TDH3* promoter (Table 1) was used. Consistent with the phylogenetic analysis (Fig. 1), the isolated metagenomics XI genes, SmmXylA1 and SmmXyl10, belonging to group of environmental Bacteroidetes, were not active in *S. cerevisiae* (JUK53a and JUK54a in Fig. 2).

**Table 1:** Strains and plasmids

Strain/Plasmid	Genotype/Property
*Saccharomyces cerevisiae*	
ATCC24860	Obtained from American Type Culture Collection
	*MATα*/*MATa*
JUK36a	ATCC 24860 derivative;
	*MATa ura3::loxP TKL1(-268, -1):: T*_*RKI1*_ *- RKI1-P*_*ADH1*_ *-T*_*RPE1*_ *-RPE1-P*_*TPI1*_ *-LoxP-T*_*XKS1*_ *-XKS1-P*_*PGK1*_ *-P*_*PDC1*_ *-TAL1-T*_*TAL1*_ *-P*_*FBA1*_ *gre3::loxP*
JUK39a	JUK36a derivative; *cyc3::loxP*
JUK50a	JUK36a derivative; *{pJFE11}*
JUK51a_1	JUK36a derivative; *{pJFX11-TDH3p}*
JUK51a_2	JUK36a derivative; *{pJFX11}*
JUK52a	JUK36a derivative; *{pJFX12}*
JUK53a	JUK36a derivative; *{pJFX13}*
JUK54a	JUK36a derivative; *{pJFX14}*
JUK61a	JUK39a derivative; *{pJFX11-TDH3p}*
JUKx11a	Obtained from the adaptive cultivation of JUK61a on xylose
JUK36a1	JUK51a_1 derivative with the loss of pJFX11-TDH3p
JUK39a1	JUKx11a derivative with the loss of pJFX11-TDH3p
36a(Bvu)	JUK36a1 derivative; *{pPY1-Bvu}*
36a(XIq)	JUK36a1 derivative; *{pPY1-*XI*q}*
36a(TAA)	JUK36a1 derivative; *{pPY1-TAA}*
36a(HGB5)	JUK36a1 derivative; *{pPY1-HGB5}*
36a(YIT)	JUK36a1 derivative; *{pPY1-YIT}*
39a(Bvu)	JUK39a1 derivative; *{pPY1-Bvu}*
39a(XIq)	JUK39a1 derivative; *{pPY1-*XI*q}*
39a(TAA)	JUK39a1 derivative; *{pPY1-TAA}*
39a(HGB5)	JUK39a1 derivative; *{pPY1-HGB5}*
39a(YIT)	JUK39a1 derivative; *{pPY1-YIT}*
*Plasmids*	
pJPPP-XK	pUC19, genomic integrative plasmid used to overexpress *Saccharomyces cerevisiae* endogenous genes *RPE1*, *RKI1*, *TAL1*, *TKL1* and *XKS1*
pJFX11-TDH3p	YEp, PTDH3-TPGK1, *Bacteroides vulgatus* XI
pJFE11	YEp, PTEF1-TCYC1
pJFX11	pJFE11, *Bacteroides vulgatus* XI
pJFX12	pJFE11, *Piromyces* sp. E2 XI
pJFX13	pJFE11, SmmXylA1 XI
pJFX14	pJFE11, SmmXylA10 XI
pPY1	pYES2, PPGK1-TCYC1
pPY1-Bvu	pPY1, *Bacteroides vulgatus* XI
pPY1-XIq	pPY1, *Orpinomyces* sp. ukk1 XI
pPY1-TAA	pPY1, *Tannerella* sp. 6_1_58FAA_CT1 XI
pPY1-HGB5	pPY1, *Alistipes* sp. HGB5 XI
pPY1-YIT	pPY1, *Paraprevotella xylaniphila* XI

### Improving xylose-fermenting background yeast strain using recombinant yeast strain harbouring *B. vulgatus* XI

**Fig. 3:**
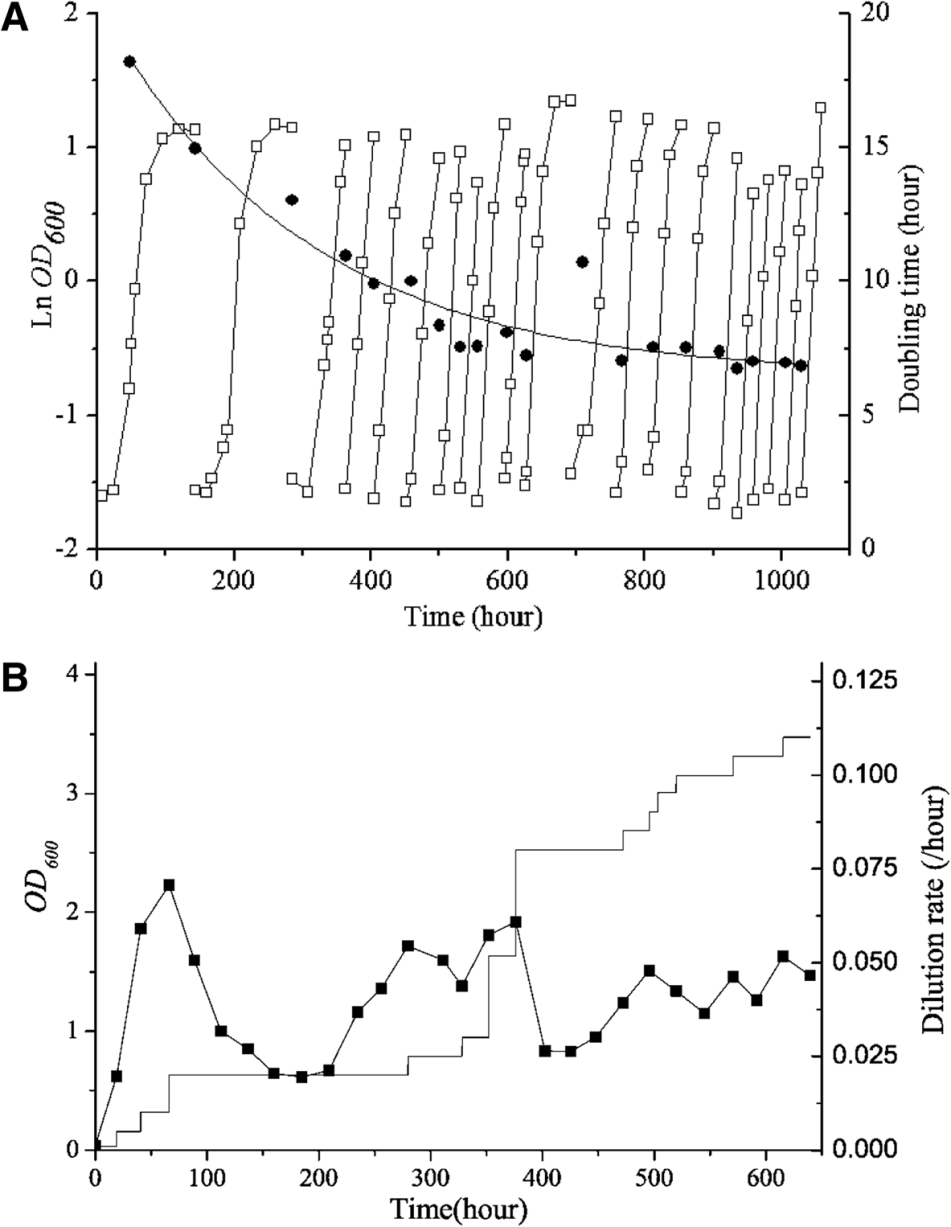
Continuous transferring cultivation of the respiration-deficient strain JUK61a (**a**) and anaerobic chemostat evolution of strain JUK51a_1 (**b**) in SMM with 20 g l^−1^ xylose at 30 °C and pH 6.0: empty square, LnOD_600_; filled circle, doubling time; filled square, OD_600_; solid line, dilution rate

Overexpressing of XI with high activities alone could not render the effective fermentation of xylose [6]. Therefore, xylose metabolic pathway in the above background *S. cerevisiae* strain was optimized by overexpressing xylulokinase (*XKS1*) and the four enzymes from non-oxidative pentose phosphate pathway, transaldolase (*TAL1*), transketolase (*TKL1*), ribose-5-phosphate isomerase (*RKI1*), and ribulose 5-phosphate 3-epimerase (*RPE1*), and disrupting the aldose reductase gene *gre3*. Host strain (JUK36a) was generated, and the recombinant yeast strains harboring *B. vulgatus* XI strains JUK51a_1 (XI controlled by *TDH3* promoter) and JUK51a_2 (XI controlled by *TEF1* promoter) exhibited aerobic growth on xylose. On the other hand, the control strain without XI genes (JUK50a) could not grow on xylose (data not shown). Adaptive laboratory evolution in xylose medium under the anaerobic conditions or using a respiration-deficient strain was shown effective to reinforce xylose catabolism to ethanol production [7, 10, 15, 21]. Therefore, before more XIs from the mammal gut Bacteroidetes cluster were tested, a respiration-deficient strain (JUK39a) was generated from JUK36a by disrupting *cyc3*, which encodes holocytochrome c synthase and attached heme to apo-cytochrome c [25], and it could not grow on glycerol (data not shown). Plasmid pJFX11-TDH3p was transformed to JUK39a to generate the respiration-deficient strain JUK61a. Strain JUK61a exhibited the delayed growth on xylose and the growth rate increased by adaptive evolution through continuous transferring cultivation in synthetic minimal medium (SMM) containing 6.7 g l^−1^ yeast nitrogen base (YNB) and 20 g l^−1^ xylose at pH 6.0 (Fig. 3a).

**Fig. 4:**
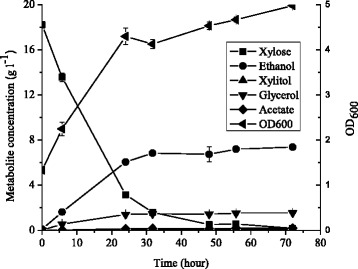
Ethanolic xylose fermentation by strain JUKx11a isolated from continuous transferring cultivation at SMM with 20 g l^−1^ xylose at 30 °C and pH. 6.0

Unlike strain JUK51a_1, for which xylose metabolism is dependent on respiration, strain JUKx11a, isolated from the continuous transferring cultivation of strain JUK61a on xylose, exhibited efficient xylose fermentation (Fig. 4). In batch aerobic cultivation, ~15 g l^−1^ xylose was consumed in 24 h, and ~6 g l^−1^ ethanol and ~1.4 g l^−1^ glycerol were produced. Ethanol yield reached 0.396 g g^−1^ consumed xylose. This strain was maintained in SMM with 20 g l^−1^ xylose and it was stable for xylose fermentation after more than 20 times of transferring. Similar results were obtained for anaerobic batch cultivation using strain JUKx11a indicating oxygen supply to this strain is not essential to this respiration-deficient strain (data not shown).

*Saccharomyces cerevisiae* strain JUK51a_1 (Table 1) was also anaerobically evolved in a chemostat by continuous diluting cultivation until its dilution rate was increased from 0.01 to 0.11 h^−1^ (Fig. 3b). A fast growing strain was isolated and the plasmid of pJFX11-TDH3p (URA3) was lost by streaking on 5-fluoroorotic acid (5-FOA) plates [26] to obtain strain JUK36a1 (Table 1). Plasmid pJFX11-TDH3p was also lost from the evolved respiration-deficient strain JUKx11a similarly to obtain strain JUK39a1 (Table 1). Both strains JUK36a1 and JUK39a1 were used as hosts for further XI testing.

### Parallel xylose utilization and cell growth testing of yeast strains expressing XI genes from mammal gut Bacteroidetes cluster

Based the background strains JUK36a1 and JUK39a1, more XIs from the mammal gut Bacteroidetes cluster (Fig. 1) were tested and compared. Beside *B. vulgatus* XI gene, XI genes from *Tannerella* sp. 6_1_58FAA_CT1, *P. xylaniphila*, *Alistipes* sp. HGB5 and *Orpinomyces* sp. ukk1 (used as a positive control) [16] were individually introduced into the evolved background strains under the control of *PGK1* promoter. The five XIs respectively have an identity of 95 % (*Orpinomyces* sp.), 82 % (*P. xylaniphila*), 81 % (*Tannerella* sp.), 81 % (*B. vulgatus*) and 76 % (*Alistipes* sp.) to the amino acid sequence of *Piromyces* sp. XI.

SMM supplied with 40 g l^−1^ xylose was used in batch cultivation of 36a and 39a strains. Aerobic cultivation was performed in 24-well plates with 600 μl media at 218 rpm and 30 °C. Duplicate wells were used for each strain. Biomass was monitored by measuring the absorbance at the wavelength of 595 nm.

**Fig. 5:**
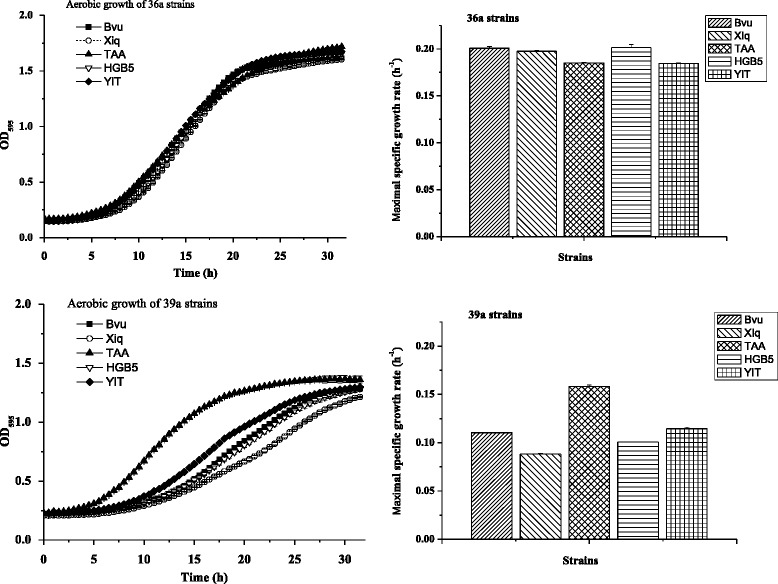
Aerobic growth and maximal specific growth rates of 36a and 39a strains in SMM with 40 g l^−1^ xylose at 30 °C and pH 6.0

The concentrations of glucose, xylose, xylitol, glycerol, acetate, and ethanol were determined using HPLC and results are listed in Fig. 5. Notably, all 36a and 39a strains expressing the above five different xylose isomerase genes grew well in the SMM containing xylose. Interestingly, all 36a strains grew almost equally fast, indicated by very similar maximal specific growth rate of about 0.2 h^−1^ (Fig. 5), with strain 36a(HGB5) containing *Alistipes* sp. HGB5 XI gene presenting the highest specific growth rate of 0.2015 h^−1^, and strain 36a(Bvu) expressing *B. vulgatus* XI gene showed very similar specific growth rate of 0.2009 h^−1^, both were slightly higher than that (0.1977 h^−1^) obtained by the control strain 36a(XIq) harboring *Orpinomyces* sp. ukk1 XI gene. Strain 36a(TAA) carrying the *Tannerella* sp. 6_1_58FAA_CT1 XI gene and 36A(YIT) containing the *P. xylaniphila* XI gene presented equal specific rate of 0.1850 h^−1^, slightly lower than other three strains. The specific rate values were much comparable to that obtained for *S. cerevisiae* expressing codon-optimized *P. ruminicola* XI gene [21]. In the latter case, a specific rate of 0.23 h^−1^ was obtained after adaptive evolution of the recombinant strain in SMM containing xylose. Apparently, the relatively high specific rates obtained for the recombinant 36a strains is attributed to the background strain, JUK36a1, which has been anaerobically evolved in xylose medium. These results demonstrated that host strain anaerobic adaptive evolution in xylose medium followed by XI gene transformation might be an effective way for the screening of novel functional xylose isomerase genes in yeast. *Orpinomyces* sp. ukk1 is a mammal gut fungus and its XI gene is very well known for its functional expression in *S. cerevisiae* [16]. The comparable performance of xylose utilization between the recombinant strains harboring the four selected XI genes from the mammal gut Bacteroidetes cluster to the control strain points out the potential of such bacteria XI genes in functional expression in *S. cerevisiae* and xylose-fermenting yeast construction.

On the other hand, cell growth rates varied among the 39a strains. Obviously, the knock-out of *cyc3* gene, which encodes holocytochrome c synthase and attached heme to apo-cytochrome c, was able to differentiate the active expression of different xylose isomerase genes. Strain 39a(TAA) harboring the *Tannerella* sp. 6_1_58FAA_CT1 XI gene demonstrated the fastest growth with 0.1584 h^−1^ specific growth rate, followed by 39a(YIT) carrying the *P. xylaniphila* XI gene with about 0.1145 h^−1^ specific growth rate and strain 39a(Bvu) containing the *B. vulgatus* XI gene with specific growth rate of 0.1104 h^−1^. Notably, the control strain 39a(XIq) expressing the *Orpinomyces* sp. ukk1 XI gene in this case displayed the lowest specific growth rate of 0.0882 h^−1^ and strain 39a(HGB5) containing *Alistipes* sp. HGB5 XI gene displayed slightly higher specific growth rate of 0.1006 h^−1^. The above results suggest that *Tannerella* sp. XI is more favorable to cell growth on xylose using the respiration-deficient strain JUK39a1 as the host. In general, 36a strains grew faster than 39a strains. The specific growth rates of 36a strains were about 0.20 h^−1^ and those for 39a strains were about 0.10 h^−1^, except strain 39a(TAA) presenting a specific growth rate of about 0.16 h^−1^. The final OD595 values of 36a strains were above 1.6, whereas those for 39a strains were below 1.4.

### Xylose fermentation by recombinant 36a and 39a strains

**Fig. 6:**
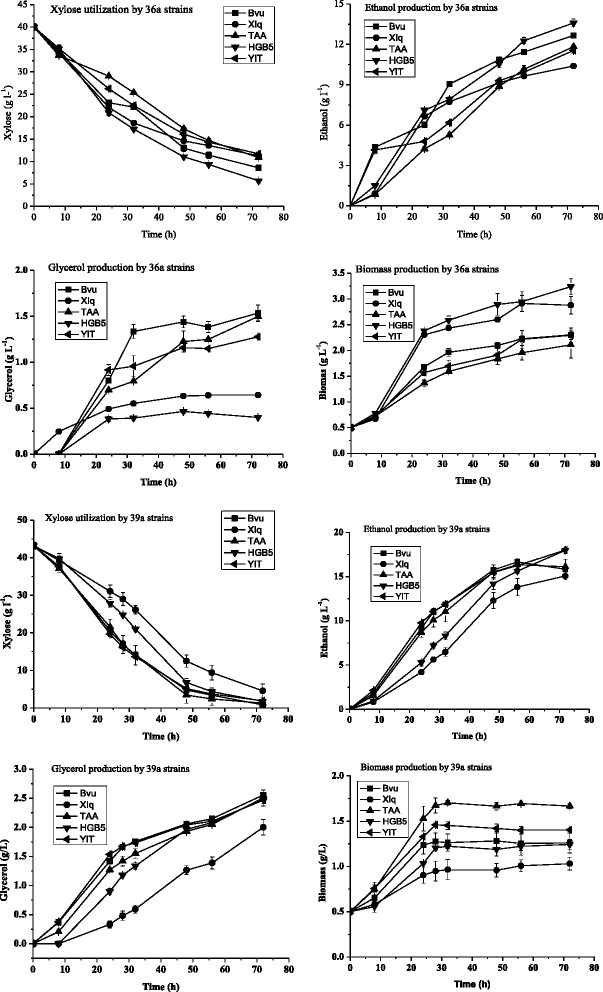
Xylose fermentation by 36a and 39a strains in SMM with 40 g l^−1^ xylose at 30 °C and pH 6.0

Better aerobic cell growth does not always correlate to better xylose fermentation. In order to identify the best xylose-fermenting recombinant yeast strains, in this part of the work, batch fermentation of recombinant 36a and 39a strains was performed in a 125 ml flask plugged with a rubber stopper with a syringe tip outlet at 30 °C and 200 rpm. SMM containing 40 g l^−1^ xylose was used in the cultivation. Interestingly, all 36a strains were able to utilize xylose as the sole carbon source and produced significant amount of ethanol (Fig. 6). This is different from the case of recombinant strains JUK51a_1 and JUK51a_2, whereby although both strains were able to grow in SMM containing xylose, no xylose-fermentation was observed. The ability of xylose-fermentation of the 36a strains was arisen from the anaerobic adaptive evolution of strain JUK51a_1 in xylose medium. Likewise, 39a strains were also able to grow in SMM containing xylose and produce ethanol, which otherwise was attributed to the anaerobic adaptive evolution of JUKx11a. For all the 36a strains tested, notably, strain 36a(HGB5) harboring *Alistipes* sp. HGB5 xylose isomerase gene was able to utilize xylose more rapidly than other strains and produced more ethanol (Fig. 6). On the other hand, for all 39a strains, strains harboring XI genes from *B. vulgatus*, *Tannerella* sp. 6_1_58FAA_CT1 and *P. xylaniphila* almost displayed almost identical rates of xylose consumption and ethanol production, with the control strain having the *Orpinomyces* sp. ukk1 XI gene showed the slowest xylose utilization and ethanol production rates (Fig. 6). Apparently, for strain 36a(HGB5) and 39a(TAA), the high rates of xylose consumption and ethanol production correlated well with the better cell growth and higher cell density. Correspondingly, the low xylose consumption and ethanol production by 39a(XIq) was due to its slow cell growth and lower cell density. However, the production of glycerol did not correlate with its cell growth. Strain 36a(HGB5) exhibited the lowest glycerol production among all the 36a strains, whereas strain 39a(TAA) displayed moderately high glycerol production among all the 39a strains.

Fermentation parameters of 36a and 39a strains at 56 h are listed in Table 2 for detailed comparison, and the average specific xylose consumption, ethanol and glycerol production rates were calculated according to Peng and his colleagues [10]. Among all the 36a strains, notably, 36a(HGB5) presented the highest ethanol titer and xylose conversion, indicating its fastest xylose utilization rate. This might be associated with its highest cell growth rate revealed by its high final biomass concentration. Correspondingly, its specific ethanol productivity was lower than the rest of strains, except the control strain 36a(XIq). Interestingly, the control strain also displayed the lowest ethanol yield of 0.365 g g^−1^, corresponding to about 72 % of the theoretical value. This arose primarily from its lower ethanol titer than the rest strains (Table 2). The main by-product for 36a strain fermentation was glycerol, with its specific production rate of about 0.02 to 0.13 g g_cdw_^−1^ h^−1^. Very minimal amount of xylitol and acetic acid were produced with their specific production rates being lower than 0.01 g g_cdw_^−1^ h^−1^ (data not shown). For the 39a strains, the highest ethanol titer was obtained by 39a(Bvu), whereas the highest xylose conversion was obtained by strain 39a(TAA) with the 2^nd^ highest ethanol titer. This is largely due to its high cell growth rate (Fig. 6) and cell density. Further analysis of the specific rates of xylose consumption and ethanol production suggest that strain 39a(HGB5) is the most potential by showing the highest rates, just below the control strain 39a(XIq). Strain 39a(Bvu) showed slightly lower specific rates of xylose consumption and ethanol production. The fact that 39a strains containing *B. vulgatus* and *Alistipes* sp. HGB5 XI genes exhibited moderately high cell growth rate and higher specific rates of xylose consumption and ethanol production recommends that these two XI genes are the most potential in xylose-fermenting yeast construction using JUK39a1 as the host. Similar to 36a strains, the main by-product for 39a strains was glycerol with specific rate of production of about 0.03 g g_cdw_^−1^ h^−1^. Production of xylitol and acetic acid was very minimal with both specific production rates being smaller than 0.01 g g_cdw_^−1^ h^−1^ (data not shown).

**Table 2:** Xylose fermentation parameters obtained in SMM with 40 g l^−1^ xylose at 56 h

Strains	Xylose conversion	Cell biomass	Ethanol	Ethanol yield	Average specific consumption/production rates		
	(%)	(g l^−1^)	(g l^−1^)	(g g^−1^)	g g_cdw_^−1^ h^−1^		
					Xylose	Ethanol	Glycerol
36a(Bvu)	71.42 ± 1.49	2.23 ± 0.02	11.43 ± 0.09	0.400 ± 0.001	0.324 ± 0.008	0.130 ± 0.001	0.012 ± 0.001
36a(XIq)	66.09 ± 0.26	2.91 ± 0.03	9.64 ± 0.06	0.365 ± 001	0.241 ± 0.001	0.088 ± 0.001	0.004 ± 0.000
36a(TAA)	63.30 ± 0.38	1.96 ± 0.02	10.15 ± 0.29	0.400 ± 0.009	0.333 ± 0.000	0.134 ± 0.004	0.013 ± 0.001
36a(HGB5)	76.68 ± 0.64	2.95 ± 0.04	12.27 ± 0.23	0.400 ± 0.004	0.261 ± 0.002	0.104 ± 0.002	0.002 ± 0.000
36a(YIT)	64.17 ± 0.05	2.22 ± 0.02	9.93 ± 0.16	0.387 ± 0.007	0.314 ± 0.000	0.121 ± 0.002	0.010 ± 0.000
39a(Bvu)	92.13 ± 0.42	1.26 ± 0.11	16.67 ± 0.08	0.419 ± 0.003	0.662 ± 0.057	0.277 ± 0.022	0.036 ± 0.003
39a(XIq)	78.26 ± 4.20	1.03 ± 0.07	13.82 ± 0.98	0.409 ± 0.014	0.721 ± 0.045	0.295 ± 0.008	0.030 ± 0.005
39a(TAA)	94.47 ± 3.82	1.67 ± 0.03	16.35 ± 0.34	0.401 ± 0.006	0.532 ± 0.011	0.213 ± 0.001	0.027 ± 0.001
39a(HGB5)	89.98 ± 2.08	1.24 ± 0.05	15.63 ± 0.30	0.402 ± 0.005	0.704 ± 0.005	0.283 ± 0.006	0.038 ± 0.001
39a(YIT)	91.39 ± 0.35	1.40 ± 0.01	16.27 ± 0.01	0.412 ± 0.005	0.586 ± 0.002	0.242 ± 0.004	0.031 ± 0.001

Strain 39a(TAA) demonstrated the lowest specific ethanol productivity (0.213 g g_cdw_^−1^ h^−1^) with the highest being 0.295 g g_cdw_^−1^ h^−1^ obtained by the control strain 39a(XIq). Interestingly, such specific ethanol productivity values for 39a strains were much higher than those reported by Peng *et al.* [10] and Hector *et al.* [21], where they were below 0.100 g g_cdw_^−1^ h^−1^, and were comparable to those obtained by 36a strains ranged from 0.104 to 0.134. Our recombinant strains were constructed by strain adaptive evolution in xylose medium followed by XI gene transformation; whereas the recombinant strains by Peng et al [10] and Hector *et al.* [21] were constructed by XI gene transformation followed by adaptive evolution in xylose medium. Much comparable cell growth (Fig. 5) and fermentation performance (Table 2) were obtained, suggesting that the approach adopted in this work is effective in rapid screening of functional XI genes and efficient in construction of xylose-fermenting yeasts.

It is worthwhile noting that consistent ethanol yield of about 0.4 g g^−1^ was obtained for all the 39a strains tested, with the highest being 0.419 g g^−1^ for strain 39a(Bvu) and the lowest being 0.401 g g^−1^ for strain 39a(TAA), corresponding to 82.2 % and 78.6 %, respectively, of the theoretical yield. The control strain 39a(XIq) in this case obtained the highest ethanol yield of 0.409 g g^−1^, corresponding to 80.2 % of the theoretical yield. The significant difference of ethanol yield for strains 36a(XIq) and 39a(XIq) confirms that respiration-deficient background strain is more favorable for the expression of fungus *Orpinomyces* sp. XI gene. Moreover, all the 39a strains consumed xylose faster and produced more ethanol than 36a strains, further proving that respiration-deficient strain is more favorable for xylose fermentation although anaerobic adaptive evolution is able to enhance it for respiration-efficient 36a strains. The favorable xylose utilization and ethanol production by 39a strains is ascribed to the anaerobic nature of these mammal gut bacteria.

## Conclusion

Xylose isomerase genes from two phylogenetic groups (mammal gut Bacteroidetes and environmental Bacteroidetes) were tested in *S. cerevisiae* strain with downstream xylose metabolic pathway optimization. Xylose isomerase from mammal gut microorganisms, *B. vulgatus, Tannerella* sp. 6_1_58FAA_CT1, *Alistipes* sp. HGB5 and *P. xylaniphila*, were actively expressed, and the two Bacteroidetes-related genes isolated from environmental metagenome did not exhibit activities. Expression of the above XI genes from the above listed mammal gut bacteria in a respiration-efficient host, *S. cerevisiae* JUK36a1, resulted in similar growth rate under aerobic conditions. On the other hand, the respiration-deficient host, JUK39a1, resulted in varied growth rates for strains harboring XI genes from different mammal gut bacteria. The expression of *Tannerella* sp. 6_1_58FAA_CT1 XI gene resulted in the fastest cell growth rate, maximal xylose conversion, and highest ethanol production under anaerobic cultivation conditions. Respiration-deficient *S. cerevisiae* strain carrying *B. vulgatus* and *Alistipes* sp. HGB5 XI genes presented moderate cell growth and relatively high specific ethanol productivity of 0.277 and 0.283 g g_cdw_^−1^ h^−1^, respectively. They are most potential in xylose-fermenting yeast construction. All respiration-deficient strains (39a strains) displayed higher rates of xylose consumption and ethanol production than 36a strains, confirming that *cyc3* gene knock-out is favorable. In addition, this work also demonstrated that host strain adaptive evolution in xylose medium followed by XI gene transformation is effective in rapid functional XI gene screening in *S. cerevisiae* and is efficient in xylose-fermenting yeast construction.

## Methods

### Cloning of xylose isomerase genes from environmental metagenomic DNA

Primers used in this work are listed in Additional file 1. Metagenomic DNA from snail manure and soil from Singapore natural forest was extracted using FastDNA® Spin Kit for Soil (MP Biomedicals) and purified using E.Z.N.A. ® Cycle-Pure Kit (Omega Bio-tek). Degenerate primers were designed based on xylose isomerase conservative region (Additional file 1). Primers MetXIconss and MetXIconsa were used to amplify partial sequences of xylose isomerase from snail manure metagenomic DNA (Smm) and soil metagenomic DNA (Som). The PCR product of partial sequences of xylose isomerase was cloned into T-vector and sequenced. The 5′ and 3′ flanking regions of xylose isomerase from metagenomic DNA were obtained through thermal asymmetric interlaced polymerase chain reaction (TAIL-PCR) using LA *Taq* DNA polymerase (TAKARA), and arbitrary degenerated primers (AD1, AD2, AD3 or AD4) and nested gene-specific primers. TAIL-PCR was performed according to the previously reported protocol [27] and the TAIL-PCR products were sequenced to generate the whole gene sequences. Primers designed based on 5′ and 3′ regions of xylose isomerase were used to amplify the full-length genes directly from metagenomic DNA.

### Plasmid construction

Strains and plasmids used in this work are listed in Table 1.

**Fig. 7:**
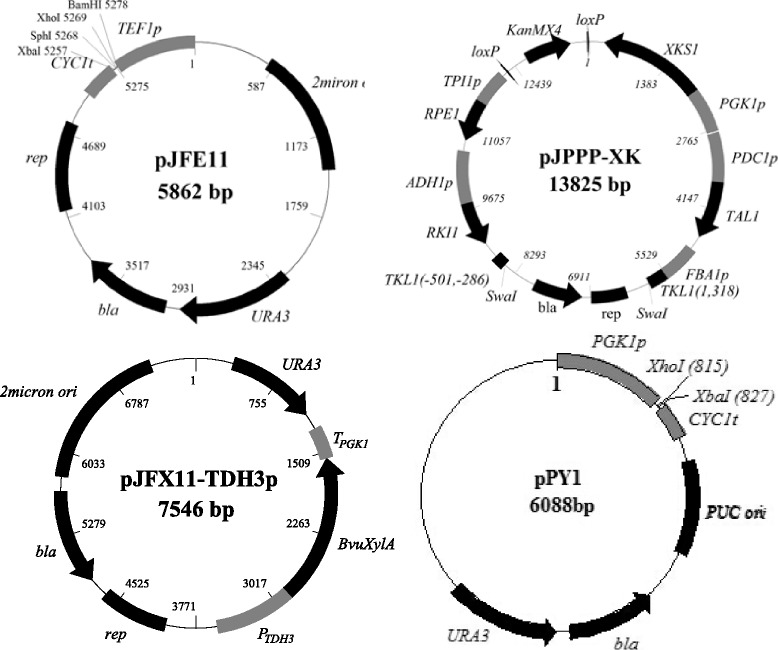
Plasmids used for the construction of xylose metabolic pathway

The gene fragments, *FBA1p-TKL1(1318)* (recombinant arm targeting to *TKL1* open reading frame, ORF), *TKL1(-501,-286)* (recombinant arm targeting to *TKL1* promoter), *PDC1p-TAL1-TAL1t*, *ADH1p-RKI1-RKI1t*, *TPI1p-RPE1-RPE1t* and *PGK1p-XKS1-XKS1p* were amplified using genomic DNA of *S. cerevisiae* ATCC 24860 and fused through overlapping extension polymerase chain reactions (PCR). G418 resistant marker *loxP-KanMX4-loxP* was amplified from plasmid pUG6. These DNA fragments were sequentially cloned into pUC19 to generate the plasmid pJPPP-XK (Fig. 7).

The coding sequence of *B. vulgates* xylose isomerase was synthesized according to the sequence under EMBL-ENA access No. ABR41556.1 and fused with *TDH3* promoter and *PGK1* terminator from *S. cerevisiae* through overlapping extension PCR to generate DNA fragment *TDH3p-BvuXylA-PGK1t*. Auxotrophic marker *URA3* was cut out from pUG72 by *Xba*I and *Nde*I. DNA fragments of *TDH3p-BvuXylA-PGK1* and *URA3* were sequentially cloned into *Sal*I/*Pst*I sites and *Spe*I/*Nde*I sites of pRS327 to generate pJFX11-TDH3p (Fig. 7). The yeast expression vector pJFE11 was constructed by replacing *GAL1* promoter of plasmid pYES2 by *TEF1* promoter. The coding sequences of *B. vulgates* XI, *Piromyces* sp. XI, Smm*XylA*1 and Smm*XylA*10 were cloned into *Bam*H/*Xho*I sites of pJFE11 (Fig. 7) between *TEF1* promoter and *CYC1* terminator to generate pJFX11, pJFX12, pJFX13 and pJFX14, respectively (Table 1).

### Strain construction

*S. cerevisiae* haploid strain was isolated from the diploid strain *S. cerevisiae* ATCC 24860. The LiAc/ssDNA/PEG solution was used for yeast transformation [28]. Disruption cassettes of *ura3*, *gre3* and *cyc3* with G418 resistant marker *loxP-KanMX4-loxP* were amplified from pUG6 [26]. These disruption cassettes were transformed into yeast to generate the strains with relevant genotypes. Xylulokinase and non-oxidative pentose phosphate pathway were overexpressed by transforming *Swa*I-digested pJPPP-XK (Fig. 7). The *KanMX4* markers for disruptions of *ura3*, *gre3* and *cyc3* and pJPPP-XK integration were separately removed by transforming a *Cre* plasmid pSH47 (*URA3*) and inducing *Cre* expression in YP galactose broth. Later on, pSH47 (*URA3*) was lost by cultivation on 5-FOA plates [26].

### Strain cultivation

Synthetic minimal media (SMM, Yeast nitrogen base, YNB, 6.7 g l^−1^, pH 6.0) supplied with glucose and/or xylose was used in batch cultivation. Aerobic cultivation was performed in 24-well plate with 600 μl medium at 218 rpm and 30 °C and cell growth was monitored by measuring the absorbance at the wavelength of 595 nm. Oxygen-limited cultivation was performed in 125 ml Erlenmeyer flasks, plugged by rubber stoppers with syringe needle outlets, with 60 ml media incubated at 200 rpm and 30 °C. Cells in late exponential-phase in SMM with 20 g l^−1^ xylose were harvested by centrifuge and inoculated into the cultivation medium for fermentation evaluation. Cell biomass was monitored by measuring the absorbance at the wavelength of 600 nm.

Adaptive transferring cultivation was performed in a 125 ml Erlenmeyer flasks, plugged by rubber stoppers with syringe needle outlets, containing 20 ml SMM with 20 g l^−1^ xylose incubated at 200 rpm and 30 °C. In each batch of adaptation, the initial OD_600_ was set as 0.2, and when OD_600_ reached about 2.5, cells were inoculated into a new batch with an initial OD_600_ of 0.2 for further adaptation. Finally, single clones were separated by streaking on the SMM xylose plate, and the biggest clone was selected and named as JUKx11a after seven day incubation.

Continuous evolutionary cultivation was performed in a 200 ml feeding bottle with 150 ml SMM supplied with 12 g l^−1^ xylose as the carbon source and a stirring magnetic bar at 30 °C, under anaerobic conditions. The cultivation was initiated by inoculating the JUK51a_1 cells cultivated in the aerobic conditions. The fresh media was continuously added through a feeding pump. Biomass was monitored by measuring the absorbance at wavelength of 600 nm. According to cell growth, the dilution rate was increased gradually from 0.01 to 0.11 h^−1^.

The concentrations of glucose, xylose, xylitol, glycerol, acetate, and ethanol were determined using HPLC with an Aminex HPX-87H ion exchange column (Bio-Rad) at 60 °C, and a refractive index detector; 5 mmol l^−1^ H_2_SO_4_ was used as the mobile phase with a flow rate of 0.6 ml min^−1^ [6]. All aerobic cultivation and xylose fermentation experiments were conducted in duplicate, and the average value with standard deviation was reported.

### Xylose isomerase activity assay

Cells were cultivated in SMM supplemented with 20 g l^−1^ xylose and were harvested at OD_600_ of 2.5. Cell-free extracts were prepared in 100 mM tris-HCl buffer (pH 7.5) using a glass bead beater and protease inhibitor cocktail set IV (Merck) was added. The xylose isomerase activity of the cell extracts was determined at ambient temperature (25 °C) using the UV-VIS Spectrophotometer 1240 (Shimadzu). The 1-ml reaction mixture contained 100 mmol l^−1^ Tris–HCl buffer (pH 7.5), 10 mmol l^−1^ MgCl_2_, 500 mmol l^−1^ xylose, 1 U of sorbitol dehydrogenase (Roche), 0.15 mmol l^−1^ NADH, and 0.05 ml of the cell extract [6]. Protein concentration was measured using the Coomassie protein assay kit (Thermo Scientific). One unit of enzyme activity was defined as the amount of enzyme required to oxidize 1 μmol of coenzyme/min, and the specific activity was expressed in units per milligram of protein.

## Additional file

Additional file 1: **Supplementary material.**

## Abbreviations

*ADH1*: Alcohol dehydrogenase
ATCC: American Type Culture Collection
CAI: Codon adaptation index
*CYC1*: Cytochrome c
*cyc3*: Holocytochrome c synthase gene
cdw: Cell dry weight
EMBL-ENA: European Molecular Biology Laboratory – European Nucleotide Archive
*FBA1*: 1,6-bisphosphate aldolase
FOA: Fluoroorotic acid
*gre3*: Aldose reductase gene
NADP^+^: Oxidized nicotinamide adenine dinucleotide phosphate
NADPH: Reduced nicotinamide adenine dinucleotide phosphate
NAD^+^: Oxidized nicotinamide adenine dinucleotide
NADH: Reduced nicotinamide adenine dinucleotide
*PDC1*: Pyruvate decarboxylase
*PGK1*: 3-phosphoglycerate kinase promoter
*RKI1*: Ribose-5-phosphate isomerase
*RPE1*: Ribulose 5-phosphate 3-epimerase
SMM: Synthetic minimal medium
TAIL-PCR: Thermal asymmetric interlaced polymerase chain reaction
*TDH3*: Glyceraldehyde-3-phosphate dehydrogenase
*TAL1*: Transaldolase
*TKL1*: Transketolase
*TEF1*: Transcription elongation factor
XI: Xylose isomerase
XKS1: Xylulokinase
XR: Xylose reductase
XDH: Xylitol dehydrogenase
YEp: Yeast episomal plasmid
