# Supplementary material for: Bacterial xylose isomerases from the mammal gut Bacteroidetes cluster function in Saccharomyces cerevisiae for effective xylose fermentation
Source: Microb Cell Fact. 2015 May 17;14:70. doi: 10.1186/s12934-015-0253-1 (PMC4436767; doi:10.1186/s12934-015-0253-1)
Supplement: Supplementary file 1 — Supplementary material. [file 12934_2015_253_MOESM1_ESM.docx]

Additional file 1

**A xylose isomerase from the mammal gut Bacteroidetes cluster functions in *Saccharomyces cerevisiae* for effective xylose fermentation**

Bingyin Peng, Shuangcheng Huang, Tingting Liu, Anli Geng *

* Correspondence Email:

Dr. Anli Geng ([gan2@np.edu.sg](mailto:gan2@np.edu.sg))

*School of Life Sciences and Chemical Technology, Ngee Ann Polytechnic, Singapore*

*Correspondence

Dr. Anli Geng

School of Life Sciences and Chemical Technology

Ngee Ann Polytechnic

535 Clementi Road

Singapore 599489

Tel: (65) 64608617; Fax: (65) 64679109

Email: gan2@np.edu.sg

**Table A1. Primers used in this work**

| Primer | | Usage | Sequence (5’->3’) |
| --- | --- | --- | --- |
| MetXIconss | | Degenerated primers based on conservative region of XI | TGGGGNGGNMGNGGNTAY |
| MetXIconsa | |  | GGRAAYTSRTCNGTRTCCCA |
| AD1 | | Arbitrary degenerate primers used in TAIL-PCR | NTCGASTWTSGWGTT (Liu et al., 1995) |
| AD2 | |  | NGTCGASWGANAWGAA (Liu et al., 1995) |
| AD3 | |  | WGTGNAGWANCANAGA (Liu et al., 1995) |
| AD4 | |  | AGWGNAGWANCAWAGG (Liu & Whittier, 1995) |
| SmmXylA1s1 | | Nested gene-specific primers used to amplify 3’ flanking sequence of Smm*XylA*1 | ATCGAGCCTAAACCCTGTGAGCCGACAA |
| SmmXylA1s2 | |  | ATCTTGAAGTTAATCACGCTACCCTTGCC |
| SmmXylA1s3 | |  | ATGGATGCCAACAGAGGCGACTATCA |
| SmmXylA1a1 | | Nested gene-specific primers used to amplify 5’ flanking sequence of Smm*XylA*1 | AGTATGACCGGCAAGGGTAGCGTGA |
| SmmXylA1a2 | |  | GCTCACAGGGTTTAGGCTCGATGAAA |
| SmmXylA1a3 | |  | CGCTAAATGCTCCTGCTCACGTTTCA |
| SmmXylA10s1 | | Nested gene-specific primers used to amplify 3’ flanking sequence of Smm*XylA*10 | TCGAGCCAAAGCCTTGTGAACCTACG |
| SmmXylA10s2 | |  | ATTCAGCTACCGTTATCAGTTTCTTGAGG |
| SmmXylA10s3 | |  | CTTCAGGTAGCAGCAGATACTGGATTG |
| SmmXylA10a1 | | Nested gene-specific primers used to amplify 5’ flanking sequence of Smm*XylA*10 | TAGTCACCACGATTAGCATCAATAGAACCCAAC |
| SmmXylA10a2 | |  | TCAGATCATATTGCCTCAAGAAACTGATAACGGTA |
| SmmXylA10a3 | |  | AATCATATTGATGTTTCGTAGGTTCACAAGGC |
| SmmXylA1fuls | | Primers used to amplify full-length Smm*XylA*1 | ATTGAACCCTGTATGACCAGCATCTACGA |
| SmmXylA1fula | |  | CGGCGACGAAATGAACATAAAGAAAC |
| SmmXylA10fuls | | Primers used to amplify full-length Smm*XylA*10 | TTCTCAGGATTGACCCAAGATCAAAACA |
| SmmXylA10fula | |  | AATGATGAGTAACCCTCATCTTCAAAGT |
| *Construction of pJPPP-XK* | | | |
| FBA1ps | | *FBA1* promoter | GCTGACGCATGCCCTAAGAAATGAATAACAATACTG |
| FBA1pa | |  | ***AGTGAATTGAGTCATTTTGAATATGTATTAC***TTGGT |
| TKL1rec1s | | *TKL1* rec arm1 | ***GTAATACATATTCAAAATGACTCAATTCACT***GACAT |
| TKL1rec1a | |  | AGCAAGCTTATTTAAATTCAGGATGACCTGGTGTTC |
| TKL1rec2s | | *TKL1* rec arm2 | TAAGAATTCATTTAAATACAGCAAACTTGCATTTTGG |
| TKL1rec2a | |  | TTAGGATCCTTTGAGCTCTTTGGTACCAGATTGCGAACGGAACG |
| PDC1ps | | *PDC1* promoter | AAAAGCATGCCATGCAAAGAGGTGGTACCCGCACG |
| PDC1pa | |  | ***TTCAGACATTTTGATTGATTTGACTG****TGTTATTTTGCG* |
| TAL1s | | *TAL1* and its terminator | ***CAGTCAAATCAATCAAAATGTCTGAA***CCAGCTCAAAAG |
| TAL1a | |  | TATCCTGCAGGTATTGCTATTGGATTTGGTGTGGGTAT |
| ADH1ps | | *ADH1* promoter | AAAGAGCTCGAAATGGTTGTCTGTTTGAGTACGC |
| ADH1pa | |  | ***ACACCGGCAGCCATTGTATATGAGATAGTTG***ATTG |
| RKI1s | | *RKI1* and its terminator | ***CAACTATCTCATATACAATGGCTGCCGGTGT***CCCA |
| RKI1a | |  | AAAGGTACCGGACAGGGTCTTGTTGAAGGATTATC |
| TPI1ps | | *TPI1* promoter | TTAGGATCCTTTCCTGCAGGTTTCTCGAGCTGATTGTGAGGGAGA |
| TPI1pa | |  | ***TTTGACCATTTTTAGTTTATGTA***TGTGTTTTTTG |
| RPE1s | | *RPE1* and its terminator | ***TACATAAACTAAAAATGGTCAAA***CCAATTATAGCTC |
| RPE1a | |  | TTTGAGCTCGAAAACGGACAAGGCACCACCC |
| PGK1ps | | *PGK1* promoter | TTCCGGATCCACTCAAGACGCACAGATATTATAAC |
| PGK1pa | |  | ***TACTGAACACAACATTGTTTTATATTTGTT***GTAAAAAG |
| XKS1s | | *XKS1* and its terminator | ***AACAAATATAAAACAATGTTGTGTTCAGTA***ATTCAG |
| XKS1a | |  | TTTCCTGCAGGTCGTTGCTGGTCGCTCACC |
| KanMX4s | | *loxP-KanMX4-loxP* | TAACTCGAGCAGCTGAAGCTTCGTACGCT |
| KanMX4a | |  | TATCCTGCAGGCATAGGCCACTAGTGGATCTG |
| *Determination of mating type* | | | |
| vMATanti |  | | TGTGGAGGCACCCAAGAAGG |
| vMATalphas |  | | GCTCGCTGAAGAATGGCACG |
| vMATas |  | | CAATGATTAAAATAGCATAGTCGGGT |
| *Gene knocking-out and verification* | | | |
| dURA3s | | Disruption cassette of *ura3* | GCTGTGGTTTCAGGGTCCATAAAGCTTTTCAATTCATCTTTCTGAAGCTTCGTACGCTG |
| dURA3a | |  | TTTTTCGTCATTATAGAAATCATTACGACCGAGATTCCCGGATAGGCCACTAGTGGATCTG |
| vdURA3s | | Verification of *ura3* disruption | ATGTGGCTGTGGTTTCAGGG |
| vdURA3a | |  | GGAGTTCAATGCGTCCATCTTTA |
| dGRE3s | | Disruption cassette of *gre3* | ATCAAATTTATGAAGCTATCAAATTAGGCTACCGTTTATCAGCTGAAGCTTCGTACGCTG |
| dGRE3a | |  | GTGAGACCTTCTTGATTACATCATTCTCGAACAGAGTTGCATAGGCCACTAGTGGATCTG |
| vdGRE3s | | Verification of *gre3* disruption | GTCTTCACTGGTTACTCTTAATAACGGTCTGA |
| vdGRE3a | |  | ACTTGGGAAGTGGTACTGCCTGGAT |
| dCYC3s | | Disruption cassette of *cyc3* | AGTATCATCCATGTCAGGGTGCCCAGTCATGCACGAGTCCAGCTGAAGCTTCGTACGCTG |
| dCYC3a | |  | GCCCTCCGTAGAAATCCAAGACGTATCTAACTTCCTTGACATAGGCCACTAGTGGATCTG |
| vdCYC3s | | Verification of *cyc3* disruption | ACGGGCAAAGATATTGGTGGG |
| vdCYC3a | |  | CGGTCCAAGAAACGGGTCAT |
| *Cloning of xylose isomerase exression cassette TDH3p-BvuXylA-PGK1t* | | | |
| TDH3ps | | TDH3 promoter | ACCCTCGAGATAAAAAACACGCTTTTTCAGTTC |
| TDH3pa | |  | ***CATTTTGTTTGTTTATGTGTGTTTA***TTCGAAACT |
| PGK1ts | | PGK1 terminator | ***TAAATTGAATTGAATTGAAATCGAT***AGATC |
| PGK1ta | |  | TTTCCTGCAGGTTTAACGAACGCAGAATTTTCGAGT |
| BvuXylAs | | *XylA* from *Bacteroides vulgatus* | ***TAAACACACATAAACAAACAAAATG***GCTACAAAAGAGTATTTTC |
| BvuXylAa | |  | ***ATCGATTTCAATTCAATTCAATTTA***GCAATACATATTTACGATG |
| TEF1ps | | *TEF1* promoter in pJFE11 | AAAGCATGCACTAGTACAATGCATACTTTGTACGTTC |
| TEF1pa | |  | CATGCTCGAGAAAGGATCCTTTGTAATTAAAACTTAGATTAGATTG |
| PiroXylAs | | *XylA* from *Piromyces* sp. E2 | TTTGGATCCAAAATGGCTAAGGAATATTTCCCACAAA |
| PiroXylAa | |  | AAAGCTCGATTATTGGTACATGGCAACAATAGCTTC |
| SmmXylA1s | | Metagenomic XylA1 |  |
| SmmXylA1a | |  |  |
| SmmXylA10s | | Metagenomic XylA10 |  |
| SmmXylA10a | |  |  |

Base pairs in bold italic fonts represent over-lap sequence involved in gene fusion.

**SEQ A1. Amino acid and nucleic acid sequences of SmmXylA1**

>FullSmmXylA1

MGQVISGEKEFFKGIGQIKFEGPKSDNPLAFRWYDENRIVAGKSMKEHFRFACAYWHSFCGTGADPFGGPSHVFPWDEKSDAVARARDKMDAAFEFITKLNLPYYCFHDVDLVDYGNDVVENERRLQAITEYAKQKQADSGVKLLWGTANVFSNPRYMNGASTNPDFHVLAHAGAQVKAALDATIALGGENYVFWGGREGYMTLLNTDMKREQEHLARFLHAAKDYARSNGFKGTFFIEPKPCEPTKHQYDYDAATVIGFLRQYDLLNDFKLNLEVNHATLAGHTFQHEMQVAADAGLLGSMDANRGDYQNGWDTDQFPNNINELAECMLVFLQSGGLKGGGINFDAKIRRNSTDMDDLFHAHIGGMDTFARALVVADNVLQQSDYLDFRKQRYSSFDNGKGSEFENGKLTLEDLRNYAAEHGEPSAKSGKQEYLENLINRYI

>FullSmmXylA1

ATGGGACAAGTAATCTCAGGAGAAAAAGAATTCTTTAAAGGCATCGGACAAATAAAGTTCGAGGGACCGAAGTCCGACAATCCTTTAGCATTTCGCTGGTATGATGAAAATCGTATTGTAGCGGGAAAATCTATGAAAGAACACTTCAGGTTCGCATGTGCCTACTGGCATTCATTTTGTGGTACCGGCGCTGATCCTTTTGGCGGGCCATCTCATGTATTTCCATGGGATGAGAAGTCAGATGCGGTAGCGCGTGCGAGGGACAAGATGGATGCTGCCTTCGAATTCATTACGAAGCTTAATCTGCCATATTACTGCTTTCACGACGTAGACTTGGTTGACTACGGAAATGACGTGGTAGAAAATGAACGCCGTTTGCAAGCTATTACCGAGTATGCTAAACAGAAGCAGGCTGATAGTGGTGTTAAGTTGCTCTGGGGTACGGCGAATGTGTTTAGCAACCCGAGATATATGAACGGTGCGTCGACTAATCCAGACTTTCATGTACTGGCGCATGCAGGTGCCCAGGTGAAGGCGGCTCTGGACGCGACCATCGCATTAGGTGGAGAAAATTATGTGTTCTGGGGAGGCCGTGAGGGATATATGACTCTGCTTAATACAGATATGAAACGTGAGCAGGAGCATTTAGCGCGATTCTTACATGCCGCTAAAGATTATGCCAGAAGTAATGGATTTAAAGGCACGTTTTTCATCGAGCCTAAACCCTGTGAGCCGACAAAACATCAGTATGATTATGATGCAGCAACAGTTATTGGTTTCTTACGTCAGTACGATCTGCTGAACGATTTTAAATTAAATCTTGAAGTTAATCACGCTACCCTTGCCGGTCATACTTTTCAACATGAGATGCAGGTCGCTGCAGATGCCGGCTTACTTGGATCTATGGATGCCAACAGAGGCGACTATCAGAATGGTTGGGATACCGATCAGTTCCCGAATAATATAAATGAACTGGCTGAATGCATGCTTGTATTCCTGCAAAGCGGAGGGCTAAAAGGCGGCGGCATTAATTTTGATGCTAAGATCCGCCGGAATTCTACTGATATGGACGATTTGTTCCATGCGCACATTGGTGGTATGGATACATTCGCACGTGCCCTGGTGGTTGCCGATAATGTTCTACAGCAGTCTGATTATCTTGACTTCCGGAAACAAAGATATTCATCTTTCGATAATGGTAAAGGCAGTGAGTTTGAAAATGGTAAACTGACGTTGGAAGATCTCAGGAATTATGCGGCCGAGCACGGCGAACCGTCAGCGAAGAGCGGAAAGCAGGAATATCTTGAAAATCTTATTAACAGATATATTTAA

**SEQ A2. Amino acid and nucleic acid sequences of SmmXylA10**

>FullSmmXylA10

MKVLTGEKEYFKNIGQIKYEGLDSDNPLAFRWYDPSKVIAGKTMEEHFKFACAYWHSFNGDGADPFGGKTHFFPWDEKSKTIDRAKDKMDAAFEFMTKLQLPYYCFHDVDLVDYSDDINENDSNLAAIVEYAKEKQKESGIKLLWGTANLFSHHRYMNGASTNPDFHVLTHAAAQVKAALDATIALKGENYVFWGGREGYMSLLNTNMKREQEHLAKFLHLSKDYARKNGFKGTFFIEPKPCEPTKHQYDYDSATVISFLRQYDLMDDFKLNIEVNHATLAGHTFQHELQVAADTGLLGSIDANRGDYQNGWDTDQFPNDLNELTEAMLIILEAGGLQGGGVNFDAKIRRNSTDPEDLFYAHIGGMDAFARALVTADNILQKSDFKKIRKDRYASFDGGKGADFENGKLTLEDLRNFAVENGEPEMRSGRQEFLENLVNRYI

>FullSmmXylA10

ATGAAAGTATTAACAGGAGAAAAAGAGTATTTTAAGAACATCGGCCAGATTAAATATGAAGGATTGGATTCTGACAATCCATTGGCATTTCGTTGGTATGATCCTTCAAAAGTAATTGCTGGAAAAACAATGGAAGAGCATTTCAAGTTTGCTTGTGCTTATTGGCATTCATTCAACGGCGATGGAGCAGATCCATTTGGTGGCAAGACTCACTTTTTTCCTTGGGATGAAAAGTCGAAAACCATTGACCGCGCCAAGGATAAGATGGATGCTGCTTTTGAATTTATGACGAAGCTTCAACTGCCATATTACTGTTTCCATGATGTGGATCTGGTGGATTATTCAGATGATATCAATGAGAATGATTCGAACCTGGCTGCCATTGTGGAATATGCGAAAGAAAAGCAAAAGGAGAGTGGAATCAAATTGTTGTGGGGTACAGCTAACCTGTTTAGTCATCATAGATACATGAACGGAGCTTCCACAAACCCTGATTTTCATGTTTTGACCCATGCTGCAGCACAGGTAAAAGCTGCTTTGGACGCAACAATTGCCTTGAAAGGAGAAAACTATGTATTCTGGGGTGGTCGTGAAGGCTATATGTCGTTATTGAACACCAATATGAAGCGTGAACAAGAACATTTAGCAAAATTCTTGCACTTGTCTAAGGATTATGCACGTAAAAATGGTTTTAAAGGAACCTTTTTTATCGAGCCAAAGCCTTGTGAACCTACGAAACATCAATATGATTATGATTCAGCTACCGTTATCAGTTTCTTGAGGCAATATGATCTGATGGATGATTTCAAATTGAATATTGAAGTTAACCATGCTACACTTGCAGGTCATACCTTTCAACATGAGCTTCAGGTAGCAGCAGATACTGGATTGTTGGGTTCTATTGATGCTAATCGTGGTGACTATCAAAATGGTTGGGATACAGATCAATTTCCAAACGATTTGAATGAACTTACAGAAGCTATGTTAATTATCCTAGAGGCAGGTGGTCTTCAAGGCGGGGGAGTTAATTTTGATGCGAAGATTAGAAGAAATTCTACTGATCCAGAAGATTTGTTCTATGCTCATATAGGAGGCATGGATGCTTTTGCACGTGCTTTGGTAACTGCTGATAATATTTTGCAGAAATCAGACTTCAAGAAAATCAGGAAAGATCGTTACGCGAGTTTCGATGGTGGTAAGGGAGCGGATTTTGAAAATGGCAAATTGACTCTTGAGGATTTAAGAAATTTTGCTGTTGAGAATGGAGAGCCTGAAATGAGAAGTGGTAGACAAGAATTCTTAGAGAATTTGGTAAACCGCTATATTTAA

References:

Liu YG, Mitsukawa N, Oosumi T, Whittier RF: **Efficient isolation and mapping of Arabidopsis thaliana T-DNA insert junctions by thermal asymmetric interlaced PCR**. Plant J 1995. 8:457-63.

Liu YG, Whittier RF: **Thermal asymmetric interlaced PCR: automatable amplification and sequencing of insert end fragments from P1 and YAC clones for chromosome walking. Genomics** 1995, 25:674-681.
